# 3D-printed polyether-ether-ketone/n-TiO_2_ composite enhances the cytocompatibility and osteogenic differentiation of MC3T3-E1 cells by downregulating miR-154-5p

**DOI:** 10.1515/med-2023-0636

**Published:** 2023-01-28

**Authors:** Zhikun Li, Yifan Li, Wei Xu, Jimin Yu, Shichao Tong, Xiangyang Zhang, Xiaojian Ye

**Affiliations:** Department of Orthopedics, Tongren Hospital, School of Medicine, Shanghai Jiao Tong University, Shanghai 200336, People’s Republic of China; Department of Clinical Medicine, Jilin Medical University, Jilin 132013, People’s Republic of China; Department of Orthopedics, Tongren Hospital, School of Medicine, Shanghai Jiao Tong University, 1111 XianXia Road, Shanghai 200336, People’s Republic of China

**Keywords:** PEEK composite, nano-TiO_2_, 3D printing, osteogenic differentiation, miR-154-5p

## Abstract

The object was to enhance the bioactivity of pure polyether-ether-ketone (PEEK) by incorporating nano-TiO_2_ (n-TiO_2_) and investigate its potential mechanism. PEEK/n-TiO_2_ composite was manufactured using a 3D PEEK printer and characterized by scanning electron microscopy (SEM), 3D profiler, energy-dispersive spectroscopy, and Fourier-transform infrared (FT-IR) analyses. Cytocompatibility was tested using SEM, fluorescence, and cell counting kit-8 assays. Osteogenic differentiation was evaluated by osteogenic gene and mineralized nodule levels. The expression of the candidate miRNAs were detected in composite group, and its role in osteogenic differentiation was studied. As a results the 3D-printed PEEK/n-TiO_2_ composite (*Φ* = 25 mm, *H* = 2 mm) was successfully fabricated, and the TiO_2_ nanoparticles were well distributed and retained the nanoscale size of the powder. The Ra value of the composite surface was 2.69 ± 0.29, and Ti accounted for 22.29 ± 12.09% (in weight), and FT-IR analysis confirmed the characteristic peaks of TiO_2_. The cells in the composite group possessed better proliferation and osteogenic differentiation abilities than those in the PEEK group. miR-154-5p expression was decreased in the composite group, and the inhibition of miR-154-5p significantly enhanced the proliferation and osteogenic differentiation abilities. In conclusion, 3D-printed PEEK/n-TiO_2_ composite enhanced cytocompatibility and osteogenic induction ability by downregulating miR-154-5p, which provides a promising solution for improving the osteointegration of PEEK.

## 1 Introduction

Polyether-ether-ketone (PEEK) is an attractive thermoplastic polymer with excellent biocompatibility, an elastic modulus similar to that of bone, favorable mechanical properties, and adequate chemical stability. Owing to such favorable properties, it is a promising alternative to metallic and ceramic orthopedic implants [1,2]. However, many *in vivo* and clinical studies indicate a less satisfying effect of PEEK implants in osteointegration. The limitation is caused by the hydrophobicity and bioinertness of PEEK, thereby inhibiting cell attachment, leading to poor bone apposition and eventually causing implant dislocation and failure [3,4]. Various techniques and materials have been developed to overcome these shortcomings. The most widely used method is surface property modification of PEEK for better bone-binding by physicochemical modification and coating deposition with bioactive ions or bone-binding materials, including titanium (Ti)/TiO_2_, hydroxyapatite (HA), zirconia, and tantalum [5]. Notably, these modifications also lead to many new problems such as degradation of the coatings and poor binding force between the coating and implant *in vivo* [6], as well as degradation of PEEK [7]. In addition to the instability of the modified surface, reproducibility and controllability of these techniques are less satisfactory [5].

Another strategy is the 3D printing of a PEEK/material composite that enables the functional groups to be distributed on the surface and inside [8,9] and circumvents all surface-modification-related issues. In contrast to traditional manufacturing, 3D printing technology is an additive manufacturing (AM) approach that can provide personalized/precise solutions to meet the clinical needs of patients [10]. Thus far, several PEEK composites such as PEEK/HA and Ti–6Al–4V/PEEK [11,12] have been manufactured by AM technology, and the Ti–6Al–4V/PEEK composites show better bone formation than commercial pure PEEK [12]. However, these composites are mechanically integrated using two independent materials. Frontier research reports that they developed a fused filament fabrication approach, also known as fused deposition modeling that realizes the direct 3D printing of extruded PEEK/HA composite filaments and evenly distributes HA particles throughout the bulk and across the surface of the native 3D-printed samples [13]. In addition, the mechanical properties of the 3D-printed-PEEK/HA composites are comparable to those of human femoral cortical bone; however, whether the bone formation ability of the composites is enhanced remains unknown.

TiO_2_ is a widely used material for surface modification, and its coating has been confirmed to considerably enhance the adhesion, proliferation, and differentiation of osteoblast cells compared to pure PEEK [14]. TiO_2_ nanoparticles (n-TiO_2_) have higher bioactivities than conventional microparticles in cell proliferation and osteointegration [15,16]. Therefore, the incorporation of n-TiO_2_ into PEEK would be a more effective way to enhance the properties of pure PEEK.

MicroRNAs (miRNAs) are a class of short non-coding RNAs (∼22 nucleotides) that have been reported as key regulators of gene expression [17]. Studies have shown that miRNAs are involved in various cellular activities, including proliferation, migration, and differentiation [17,18]. Osteogenesis is regulated by several miRNAs [19], and coating with TiO_2_ alters the expression of miR-17 and miR-21 in MC3T3-E1 cells [20]. In addition, miR-154-3p, miR-154-5p, and miR-770-5p expression levels are considerably altered in human adipose-tissue-derived stem cells (hASCs) cultured or induced in the TiO_2_ nanotube [21]. This observation indicated that these miRNAs may be associated with the superior properties of TiO_2_.

In the present study, we aimed to enhance the cytocompatibility and osteogenic induction activity of pure PEEK by incorporating n-TiO_2_ and investigate the role of miRNA in this process. We intended to manufacture the PEEK/n-TiO_2_ composite by 3D printing technology and characterized the same using scanning electron microscopy (SEM), 3D profiler, elemental, and Fourier-transform infrared (FT-IR) analyses. The cytocompatibility and osteogenic induction ability of the composite were then evaluated *in vitro*. Next, five candidate miRNAs (miR-17, miR-21, miR-154-3p, miR-154-5p, and miR-770-5p) were verified, and miR-154-5p was found to be involved in the improved cytocompatibility and osteogenic induction ability of the 3D-printed composite.

## 2 Materials and methods

### 2.1 Preparation of the 3D-printed PEEK/n-TiO_2_ composite

First, the TiO_2_ nanopowder (Sigma-Aldrich; Merck KGaA, Darmstadt, Germany) was mixed with PEEK powder (VESTAKEEP^®^ 2000FP; Evonik, Essen, Germany) to obtain mixed powders with different n-TiO_2_ contents (0 and 30 wt%). The powders were then processed using a co-rotating twin-screw extruder (PolyLab HAAKE Rheomex OS PTW16, *D* = 16 mm, *L*/*D* = 40; Thermo Fisher, USA) to obtain continuous PEEK/n-TiO_2_ filaments (*Φ* = 1.75 ± 0.10 mm). The parameters of the two types of powder are listed in Table 1.

**Table 1:** Parameters of PEEK and TiO_2_ powders

**Parameters**	**PEEK powder**	**TiO**_**2**_ **nanopowder**
Powder size (nm)	50,000	21
Density (g/mL)	1.3	4.26
Mw	328	79
Melting point (℃)	340	1,850
Melt viscosity (Pa s)	350	/
Purity (%)	99.9	99.5

Next, 3D models of the PEEK and PEEK/n-TiO_2_ composite samples (*Φ* = 25 mm, *H* = 2 mm) were designed using CAD modeling software (Mimics; Materialize, Belgium). Subsequently, the filaments were assembled using a 3D PEEK printer (Medvance, Shanghai, China), melted at 450°C, injected into nozzles (*Φ* = 0.4 mm), and deposited layer-by-layer (0.1 mm) following the designed program. The printing speed was 10 mm/s, and the plate and chamber temperatures were maintained at 260 and 220°C, respectively. Several hours later, PEEK (tensile modulus = 6.15 GPa) and a PEEK/n-TiO_2_ composite (30 wt%, tensile modulus = 4.15 GPa) were successfully manufactured.

The tensile moduli of the samples were determined using the standard tensile test method. First, a standard tensile test sample (ISO527-2:1993 1BA) (*n* = 1) was manufactured using a 3D printer with the aforementioned printing parameters. Tensile testing was conducted by the Weipu Technology Group (Shanghai, China) following the standard tensile test method (ISO527-2:1993). An electronic universal testing machine (Instron 5969, Canton, MA, USA) was used for tensile testing. The test speed was 1 mm/min, and the maximum loading force was 50 kN. The tensile modulus was calculated according to a previous study [22].

### 2.2 Characterization of the PEEK/n-TiO_2_ composite

To study the microstructure of the samples, they were pre-treated with gold sputtering and then observed using a scanning electron microscope (Zeiss Sigma 300, Oberkochen, Germany). Macromorphologies of the two samples (*n* = 1) were photographed using a conventional camera. Energy-dispersive spectroscopy (EDS; Zeiss) was used to analyze the surface elements (C, O, and Ti) of the samples (*n* = 1). Two different square areas of each sample were randomly selected for the elemental analysis.

*Surface roughness measurement*: Surface roughness of the samples (*n* = 1) was analyzed using a 3D profile measurement laser microscope (Bruker Contour GTK, Karlsruhe, Germany). The mean surface roughness or arithmetic mean deviation of the profile (Ra), root mean square of the profile (Rq), and maximum peak-to-valley height of the profile (Rz) were measured. The surface roughness of each sample was measured twice in different areas. The measurement area was approximately 627 μm × 470 μm.

*FT*-*IR analysis*: FT-IR spectra of the samples (*n* = 1) were recorded with a Bruker Vertex 70v (Bruker, Billerica, MA, USA) using wavenumbers ranging from 1,000 to 4,000 cm^−1^ with a resolution of 2 cm^−1^, averaging 128 scans.

### 2.3 Cytocompatibility evaluation

*Cells and cell culture*: Mouse pre-osteoblast (MC3T3-E1) cells, purchased from Cell Bank of Chinese Academy of Sciences (Shanghai, China), were cultured in α-minimum essential medium (Gibco) supplemented with 10% fetal bovine serum (Gibco) and 1% penicillin/streptomycin (Gibco) at 5% CO_2_ and 37°C.

*SEM detection*: PEEK and PEEK/n-TiO_2_ composite samples were placed into a tissue culture-treated six-well cell culture plate, and the MC3T3-E1 cells were seeded on the surface of the samples (*n* = 1) at a density of 8 × 10^4^ cells/well. After a 3-day culture, the samples were washed thrice with phosphate buffered saline (PBS) (Servicebio, Wuhan, China) and immersed in 2.5% paraformaldehyde (Servicebio) for 1 h at room temperature. Different concentrations of alcohol (Servicebio) were then used for gradient dehydration. Subsequently, the samples were left to dry overnight in a fume hood and then, sputter-coated with Au. The cells attached to the surface were observed using SEM imaging (Zeiss).

*Fluorescence detection*: Cells seeded on the surface of the samples (*n* = 1) were fixed with 4% paraformaldehyde (Servicebio), washed thrice with PBS, and incubated with 5 μM 1,1′-dioctadecyl-3,3,3′,3′-tetramethylindocarbocyanine perchlorate (Beyotime, Shanghai, China) for 20 min. The cell morphology was observed using a laser scanning confocal microscope (Leica, Wetzlar, Germany).

Cell Counting Kit-8 (CCK-8 kit; Dojindo Laboratories, Kumamoto, Japan) was used to detect cell proliferation. Briefly, MC3T3-E1 cells (4 × 10^4^ cells/well) were seeded on the surface of the PEEK and PEEK/n-TiO_2_ composite samples (*n* = 3). After the cells were cultured for 1, 4, and 7 days, 200 µL of CCK-8 reagent (Dojindo Laboratories) was added to each well and incubated for another 3 h. Next, the samples were removed and the optical density (OD) at 450 nm of each well was measured using a spectrophotometer (BioTek Instruments Inc., Winooski, VT, USA). Cell proliferation was calculated using the following formula: Proliferation rate = Experimental OD/Control OD.

### 2.4 qPCR analysis of osteogenic genes expression

*Osteogenic induction*: Cells (1.5 × 10^5^ cells/well) were seeded on the surface of the PEEK and PEEK/n-TiO_2_ composite samples (*n* = 3). After the cell density reached 80%, 50 µg/mL ascorbate-2 phosphate (Sigma-Aldrich; Merck KGaA), 100 nM dexamethasone (Sigma-Aldrich), and 10 mM β-glycerophosphate (Sigma-Aldrich) were added for osteogenic induction.

*qPCR*: Total RNA of the cells induced in osteogenic medium for 7 days was extracted and purified using a TaKaRa MiniBEST Universal RNA Extraction Kit (TAKARA, Dalian, China) following the manufacturer’s instructions. Complementary DNA (cDNA) was synthesized using a ReverTra Ace qPCR RT Kit (TOYOBO, Osaka, Japan) following the manufacturer’s protocol. Next, cDNA (1 µL) was mixed with 1 µL of each primer, 10 µL of SYBR Green (Takara, Dalian, China), and 7 µL of deionized water to obtain a final volume of 20 µL. The reaction was performed at 95°C for 5 min, followed by 42 cycles at 95°C for 5 s, and 60°C for 1 min. Glyceraldehyde-3-phosphate dehydrogenase (GAPDH) was used as an internal reference gene, and the relative expression of related genes was calculated using the 2^−ΔΔCT^ method. For miRNAs, U6 was used as the internal control. The primer sequences are listed in Table 2.

**Table 2:** Primer sequences used for qPCR

Genes/miRNAs	**Forward (5**′**–3**′)	**Reverse (5**′**–3**′)
OCN	CTGCAAAGGTTGGCAGAGATG	CCACGGAAACGCTCTAGGAA
OPN	ATCTCACCATTCGGATGAGTCT	TGTAGGGACGATTGGAGTGAAA
COL1	GCTCCTCTTAGGGGCCACT	ATTGGGGACCCTTAGGCCAT
RUNX2	GACTGTGGTTACCGTCATGGC	ACTTGGTTTTTCATAACAGCGGA
ALP	CCAACTCTTTTGTGCCAGAGA	GGCTACATTGGTGTTGAGCTTTT
miR-17	AGGCCCAAAGTGCTGTTCGT	GTGCAGGGTCCGAGGT
miR-21	CTCGCTTCGGCAGCACA	GCCGCTAGCTTATCAGACTCAACA
miR-154-3p	TAGGTTATCCGTGTTG	ATCCAGTGCAGGGTCCGAGG
miR-154-5p	CTCGAGGCTTCTAAGCTGGGAACTTTGTC	ACTGAATTCCGCTTGTCTTGGACATATGGCACT
miR-770-5p	ATCCAGT GCGTGTCGTG	TGCTTCCAGTA CCACGTGTC
U6	TGCGGGTGCTCGCTTCGCAGC	CCAGTGCAGGGTCCGAGGT
GAPDH	AGGTCGGTGTGAACGGATTTG	GGGGTCGTTGATGGCAACA

### 2.5 Western blot analysis of the protein expression level of osteogenic genes

Cells seeded on the surface of PEEK and PEEK/n-TiO_2_ composite samples (*n* = 3) were induced with osteogenic medium for 7 days, and then the total protein was collected. Briefly, cells were lysed with radio-immunoprecipitation assay buffer (Beyotime, Shanghai, China) supplemented with a protease inhibitor cocktail (MCE, NJ, USA), and the protein concentration was determined using a BCA kit (Beyotime) following the manufacturer’s instructions. Subsequently, the protein sample was mixed with loading buffer (Beyotime) and denatured at 100°C for 10 min. The next day, the samples were separated using 10% SDS-PAGE and transferred to a PVDF membrane (Merck, Darmstadt, Germany). After blocking with fat-free milk for 2 h at room temperature, the membrane was cut into several pieces according to the potential MW of the protein, followed by an incubation with the corresponding primary antibody overnight at 4°C. Thereafter, the membranes were incubated with a horseradish peroxidase (HRP)-conjugated secondary antibody at room temperature. Finally, the signal was visualized using a Chemiluminescent Imaging System (Tanon, Shanghai, China) after signal enhancement using an ECL kit (Tanon), and GAPDH was used as the internal control. The following antibodies were used: OCN (#A20800, 1:1,000), OPN (#A1499, 1:1,000), COL (#A1352, 1:1,000), RUNX2 (#A2851, 1:1,000), ALP (#A4304, 1:1,000), GAPDH (#AC001, 1:1,000), and a HRP-conjugated secondary antibody (#AS014, 1:2,000).

### 2.6 Alizarin red S (ARS) staining

After the cells seeded in the samples (*n* = 3) were induced in the osteogenic medium for 14 days, the samples were washed thrice with PBS (Servicebio), immersed in 2.5% paraformaldehyde (Servicebio) for 30 min, and then incubated with 0.1% ARS stain (Solarbio, Beijing, China) for 10 min. Next, 100 mM cetylpyridinium chloride (Sigma-Aldrich) was added to each well to dissolve the stained nodules, the solution was collected, and the OD of each sample was measured at 562 nm. Relative mineralization level (OD_experimental_/OD_control_) was used to indicate the osteogenic level.

### 2.7 Cell transfection

Cells were seeded into a six-well plate and the inhibitor sequences were transfected using Lipofectamine 3000 (Invitrogen, CA, USA) following the manufacturer’s instructions when the cell confluence reached ∼80%. After 48 h of transfection, the cells were harvested for qPCR analysis and cell function experiments. The miR-154-5p inhibitor (5ʹ-UAGGUUAUCCGUGUUGCCUUCG-3ʹ) and negative control inhibitor (inhibitor NC; 5′-TAACACGTCTATACGCCCA-3′) were synthesized by RiboBio (Guangzhou, China).

### 2.8 Statistical analysis

Quantitative data are expressed as mean ± standard deviation (SD) from at least three independent experiments. All analyses were performed using SPSS 16.0 (SPSS Inc.). The Student’s *t*-test was used to evaluate the statistical significance of the differences between the two groups. Statistical significance was set at *P* < 0.05.

## 3 Results

### 3.1 Morphological features of the 3D-printed PEEK/n-TiO_2_ composite

**Figure 1:**
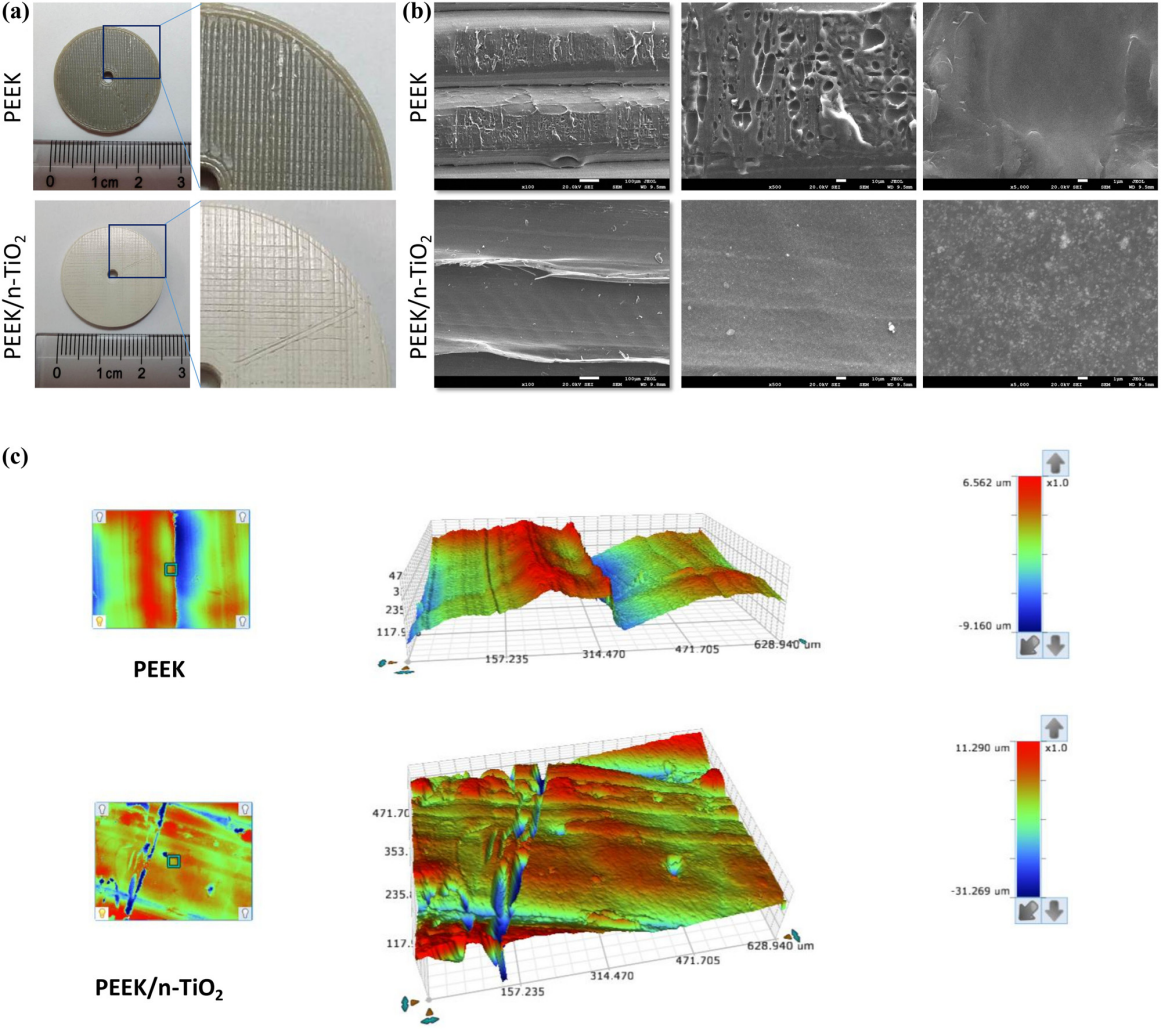
Morphological features of 3D-printed PEEK/n-TiO_2_ composite. (a) Macromorphological of 3D-printed PEEK and PEEK/n-TiO_2_ composite samples. (b) SEM was used to observe the microstructure of the two samples. Scales: 100, 10, and 1 µm (from left to right). (c) 3D profiler was used to detect the surface roughness of two samples.

As shown in Figure 1a, the circular samples shared a size of *Φ* = 25 mm and *H* = 2 mm, and the PEEK/n-TiO_2_ composite was much whiter than the PEEK control. The local enlargement images indicated that the surface lines of the two samples were both crisscross in a regular manner, indicating the moving trajectory of the nozzle during the printing process (Figure 1a, right).

SEM imaging showed that the microstructure of the PEEK sample was consistent with its macroscopic morphology, and the surface of PEEK was less smooth, with many irregular pores (Figure 1b). The bottom microstructure of the PEEK/n-TiO_2_ composite was relatively smooth, and uniformly distributed powders without significant aggregation (most particles were less than 1 μm) could be clearly observed under a magnification of 5,000 (Figure 1b, right).

**Figure 2:**
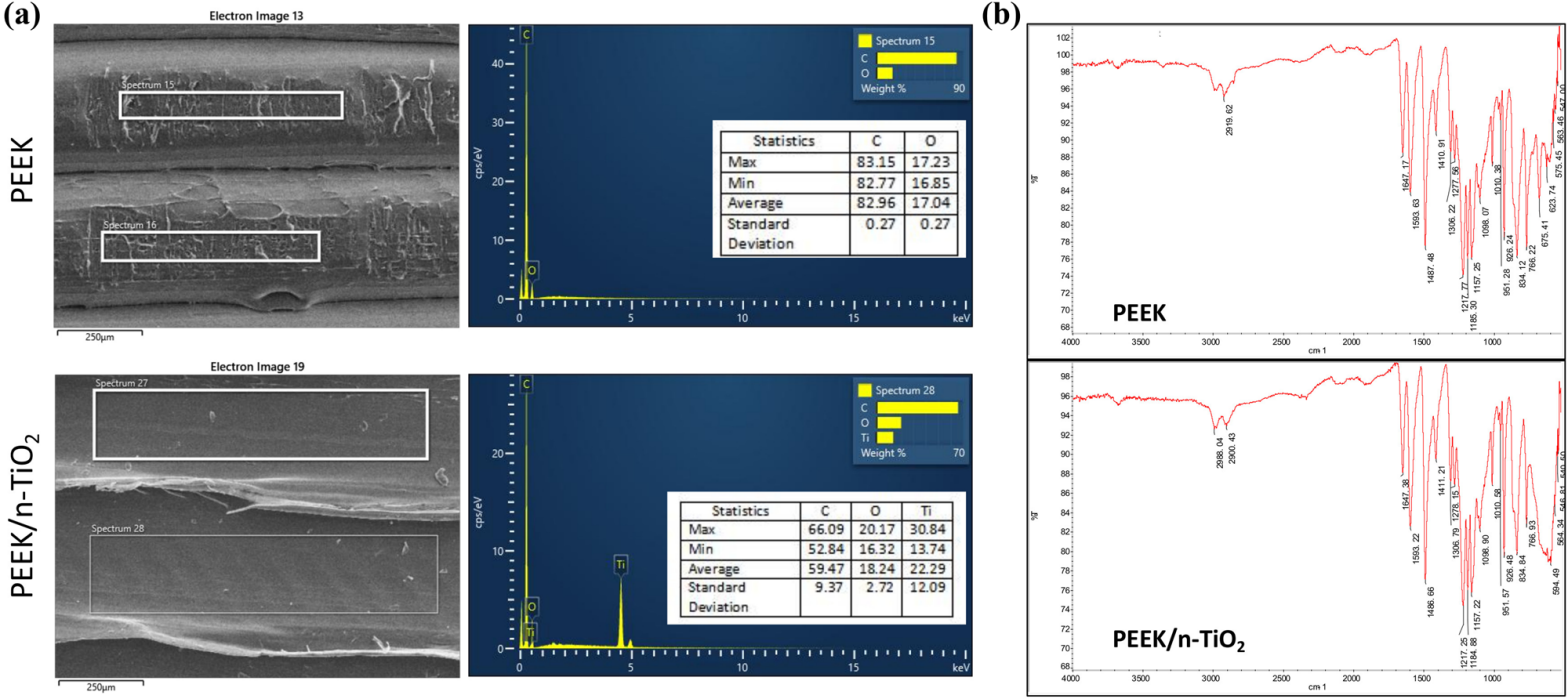
Elemental and FT-IR analyses of PEEK and PEEK/n-TiO_2_ composite samples. (a) Randomly selected areas used for EDS analysis (left, scale: 250 µm) and results of dispersive spectroscopy analysis (right). (b) FT-IR analysis of characteristic absorption peaks of two samples.

3D profiler analysis showed that the outlines of the two samples were consistent with the corresponding SEM results (Figure 2b). The Ra values of the two samples were similar, while the Rq and Rt values of the composite samples were much higher than those of the PEEK sample (Table 3). This indicated a rougher surface of the composite.

**Table 3:** Surface roughness of PEEK and PEEK/n-TiO_2_ composite

	**PEEK**			**PEEK/n-TiO**_**2**_		
	Area 1	Area 2	Mean ± SD	Area 1	Area 2	Mean ± SD
Ra (μm)	2.147	2.88	2.51 ± 0.52	2.486	2.893	2.69 ± 0.29
Rq (μm)	2.715	3.686	3.20 ± 0.69	3.579	4.748	4.16 ± 0.83
Rt (μm)	15.722	25.758	20.74 ± 7.10	42.558	64.645	53.60 ± 15.62

### 3.2 Elemental and FT-IR analyses of PEEK/n-TiO_2_ composite

EDS analysis showed that the surface of the PEEK sample comprised C (82.96% by weight) and O (17.04% by weight), and the SD values of the two elements were both less than 0.3 (Figure 2a). In the PEEK/n-TiO_2_ composite sample, nearly 60% was C, and the other two elements, O and Ti, accounted for 18.24 ± 2.72% and 22.29 ± 12.09%, respectively (Figure 2a), indicating the addition of TiO_2_ nanopowder before printing. Notably, the SD values of the three elements varied significantly from 2.72 to 12.09; hence, fully mixing the two different powders at the microlevel was a challenge and requires further research.

The FT-IR analysis indicated that the characteristic peaks of the two samples were similar, with several typical peaks of PEEK. For instance, the peak at 1,647 cm^−1^ indicated C═O stretching, and the peaks at 1,593 and 1,487 cm^−1^ were attributed to C−C stretching of the aromatic rings (Figure 2b). Furthermore, the peaks at 1,217 and 1,098 cm^−1^ indicate the C−O−C stretching vibrations of the aromatic ether bond. Compared to the PEEK spectrum, there was an intense absorption band at ∼594 cm^−1^ in the composite spectrum (Figure 2b), indicating Ti–O–Ti stretching.

### 3.3 PEEK/n-TiO_2_ composite showed better cytocompatibility and osteogenic induction ability

**Figure 3:**
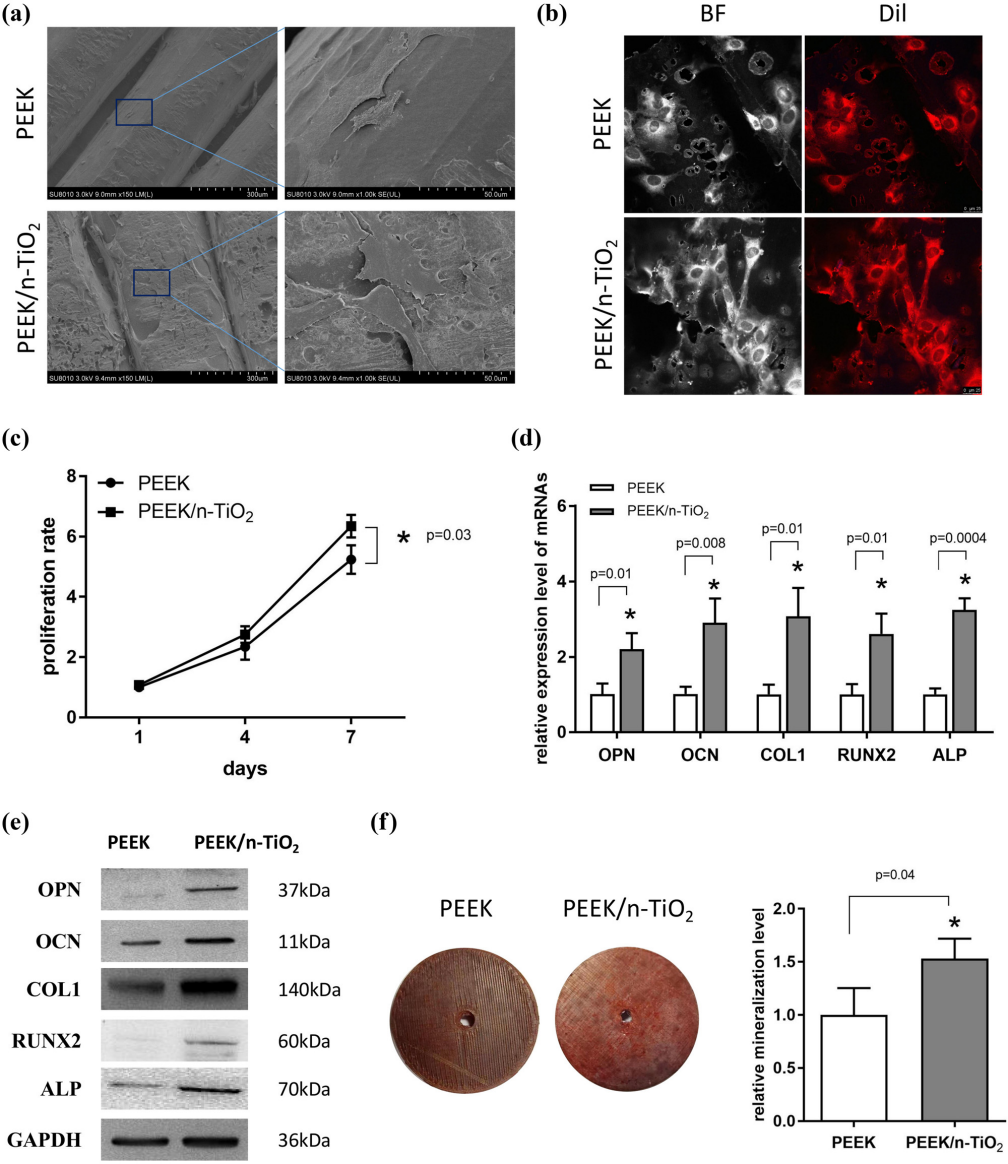
PEEK/n-TiO_2_ composite showing better cytocompatibility and osteogenic induction ability. (a) MC3T3-E1 cells were cultured on the surface of the PEEK and PEEK/n-TiO_2_ composite samples for 3 days; subsequently, SEM imaging was employed to observe cell morphology and distribution. Scales: 300 µm (left) and 50 µm (right), respectively. (b) MC3T3-E1 cells were cultured on the surface of the two samples for 3 days and stained with Dil; then, laser scanning confocal microscope was used to observe cell morphology and distribution. BF: bright field, Scale: 25 µm. (c) CCK-8 was used to detect the proliferation of cells cultured on the surface of the two samples for 1, 4, and 7 days. (d and e) qPCR and western blot were used to detect the mRNA and protein expression levels of OPN, OCN, COL1, RUNX2, and ALP, after the cells seeded on the surface of the two samples were induced with osteogenic medium for 7 days. (f) ARS staining was used to detect the mineralization level after the cells cultured on the surface of the two samples experienced the osteogenic induction for 14 days. * *P* < 0.05.

After MC3T3-E1 cells were cultured on the surfaces of the two samples for 3 days, SEM was employed to detect cytocompatibility. As shown in Figure 3a, the cells were well attached on both the materials. The cells in the PEEK group were slender in morphology, and the number of attached cells in the PEEK/n-TiO_2_ group was more than that in the control group (local vision-based observation), indicating a potentially higher cytocompatibility of the composite. In addition, the surface of the PEEK/n-TiO_2_ composite, with many bumps and hollows, was much rougher than the PEEK surface. Fluorescence analysis indicated that some cells in the PEEK/n-TiO_2_ group also showed a slender shape (Figure 3b), consistent with the SEM results. The surfaces of the samples were not flat, and the pores were visible under a bright field (Figure 3b), which also caused a higher background under the fluorescent field. Subsequently, a cell proliferation assay was performed to quantitatively compare cytocompatibility between the two materials. The results presented in Figure 3c indicate that there was no significant difference between the two groups in the proliferation rate on the fourth day. After an additional 3 days of culture, the proliferation rate of cells in the PEEK/n-TiO_2_ group was significantly enhanced compared to that in the PEEK group, suggesting that the PEEK/n-TiO_2_ composite had better cytocompatibility than pure PEEK (Figure 3c, *P* < 0.05).

To investigate the role of the PEEK/n-TiO_2_ composite in the osteogenic differentiation of MC3T3-E1 cells, qPCR was used to detect the expression levels of osteogenic genes. As shown in Figure 3d, the qPCR results showed that the mRNA levels of OPN, OCN, COL1, RUNX2, and ALP were significantly increased in the PEEK/n-TiO_2_ group compared to the PEEK group (*P* < 0.05) after the cells were induced in the osteogenic medium for 7 days. Western blotting results also indicated an increase in the protein levels of the five osteogenic markers (Figure 3e). Subsequently, the cells were further induced for another 7 days, and ARS staining was used to evaluate the mineralization level. As a result, the cells seeded on the surface of the PEEK/n-TiO_2_ composite showed a higher mineralization level than the cells on the PEEK surface (Figure 3f, *P* < 0.05). These results indicated that the addition of n-TiO_2_ significantly enhanced the osteogenic differentiation of MC3T3-E1 cells compared to pure PEEK.

### 3.4 Inhibition of miR-154-5p promotes the proliferation and osteogenic differentiation of MC3T3-E1 cells

**Figure 4:**
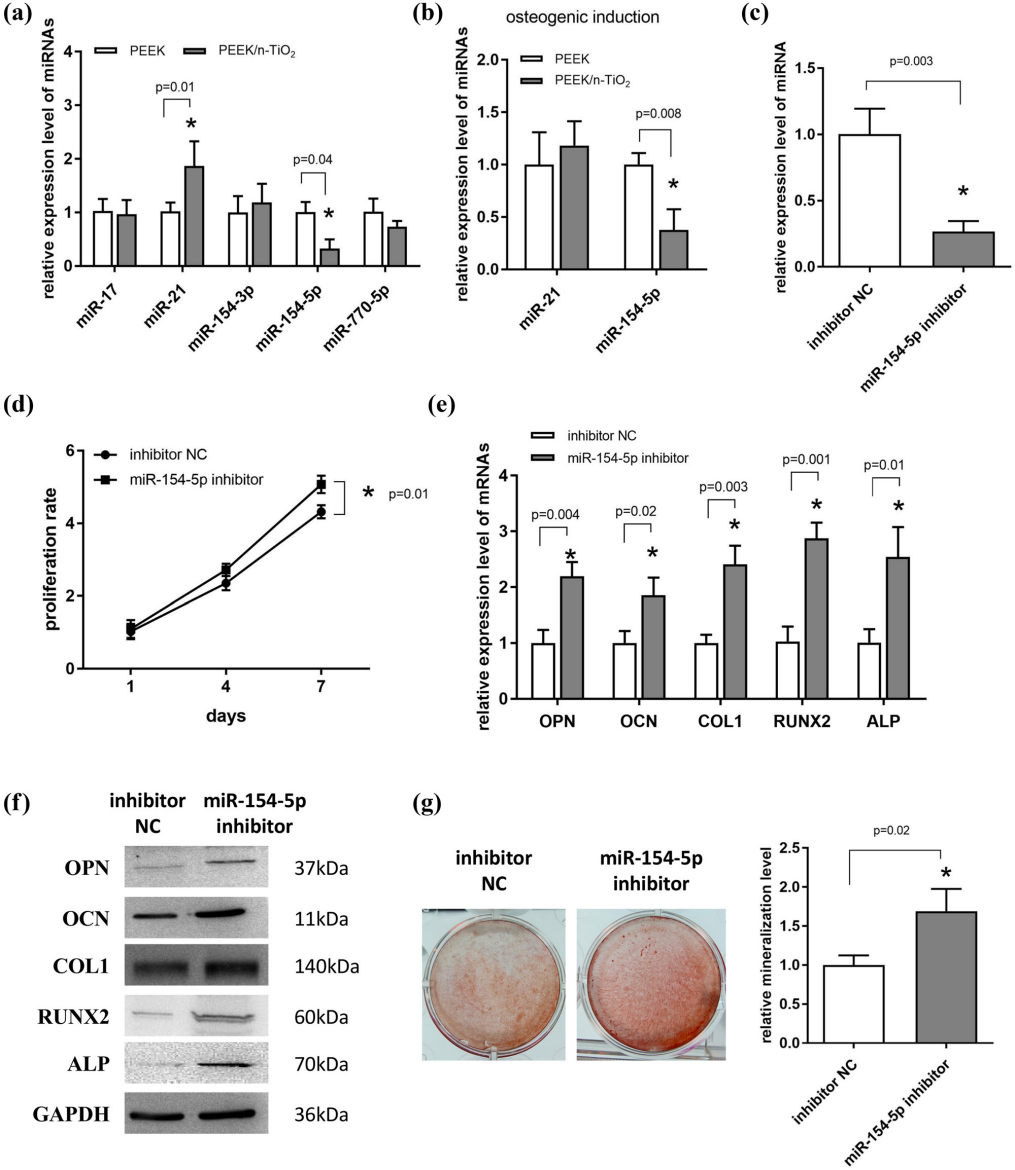
Inhibition of miR-154-5p promotes the proliferation and osteogenic differentiation of MC3T3-E1 cells. (a) Expression levels of five miRNAs were detected by qPCR after MC3T3-E1 cells were seeded on the surface of the PEEK and PEEK/n-TiO_2_ composite samples and underwent osteogenic induction for 7 days. (b) Expression levels of the two miRNAs were detected by qPCR after cells were induced on the surface of the two samples for 7 days. (c) qPCR validation of the interference effect of the miR-154-5p inhibitor. (d) CCK-8 was used to detect the proliferation potency of cells infected with the miR-154-5p inhibitor for 1, 4, and 7 days. (e and f) qPCR and western blotting were used to detect the mRNA and protein expression levels of OPN, OCN, COL1, RUNX2, and ALP after the cells were infected with the miR-154-5p inhibitor and underwent osteogenic induction for 7 days. (g) ARS staining was used to detect the mineralization level after the cells infected with the miR-154-5p inhibitor experienced osteogenic induction for 14 days. *P < 0.05.

To screen the miRNAs involved in the enhanced properties of the PEEK/n-TiO_2_ composite, the expression level of five candidates (miR-17, miR-21, miR-154-3p, miR-154-5p, and miR-770-5p) was detected, and two of them (miR-21 and miR-154-5p) were significantly altered in the PEEK/n-TiO_2_ group (Figure 4a, *P* < 0.05). Next, MC3T3-E1 cells were induced on the composite surface for 7 days, and only miR-154-5p was reduced significantly in the PEEK/n-TiO_2_ group compared to the PEEK group (Figure 4b, *P* < 0.05). Therefore, miR-154-5p may be related to the better cytocompatibility and osteogenic induction ability of the PEEK/n-TiO_2_ composite.

To confirm the role of miR-154-5p, MC3T3-E1 cells were transfected with its inhibitor, and qPCR validation indicated that its expression level was significantly inhibited (Figure 4c). Further CCK-8 detection showed that the inhibition of miR-154-5p promoted the proliferation of MC3T3-E1 cells (Figure 4d, *P* < 0.05). After 7 days of induction, the mRNA levels of five osteogenic markers in the miR-154-5p inhibitor group were all significantly enhanced compared to the inhibitor NC group (Figure 4e, *P* < 0.05). Western blotting results indicated that the protein levels of the five osteogenic markers were also increased in the miR-154-5p inhibitor group (Figure 4f). As shown in Figure 4g, cells transfected with the miR-154-5p inhibitor exhibited higher mineralization levels compared to the inhibitor NC group (*P* < 0.05). These results indicate that the inhibition of miR-154-5p promotes the proliferation and osteogenic differentiation of MC3T3-E1 cells.

## 4 Discussion

3D printing technologies not only overcome many restraints in traditional manufacturing techniques but also avoid various complications that arise during the surface modifications for property enhancements of the original materials. In this study, for the first time, we prepared a 3D-printed PEEK/n-TiO_2_ composite with a better cytocompatibility and osteogenic induction ability with MC3T3-E1 cells, providing a new strategy to improve the osteointegration of PEEK.

The introduction of functional materials significantly enhances the bioactivities of pure PEEK; however, the mechanical properties of the composite PEEK changed significantly with the decrease in PEEK content. For instance, Hughes and Grover prepared several calcium sulfate (CaS)–PEEK composites through compression molding, in which the CaS content varies from 20 to 80 wt%. They observed that the addition of 20 wt% CaS exhibited the best mechanical performance, while the mechanical parameters of the 80 wt% CaS group decreased by 50–80% compared to the 20 wt% CaS group [23]. Therefore, maintaining the dominant proportion of PEEK (e.g., >60 wt%) is crucial so that the excellent mechanical properties of PEEK can be preserved. A recent study showed that the incorporation of HA (5–30 wt%) enhanced the mechanical properties of pure PEEK, and the increased mechanical behaviors are still in line with those of human femoral cortical bone [13]. In addition, the Young’s modulus of the four TiO_2_–PEEK/PEI blends (1–8 wt% of TiO_2_) varies from ∼4.2 to ∼5.5 GPa [15], which is also comparable to that of cancellous bone (3.78 GPa) and cortical bone (14.64 GPa) [22]. In this study, we initially prepared three types of PEEK/n-TiO_2_ composites (10, 20, and 30 wt% n-TiO_2_). As expected, the tensile modulus of the three printed composites were relatively close, varying from 4.15 to 5.68 GPa (data not shown). Therefore, the composites with the highest n-TiO_2_ content was selected for further study.

Compared with conventional TiO_2_ microparticles, nanoparticles possess higher bioactivities in cell adhesion, proliferation, and osteointegration [16,24]. For instance, both osteoblasts and chondrocytes, when exposed to TiO_2_ nanofillers, exhibit an expanded morphology and increased proliferation ability compared with cells exposed to microparticles [25]. Another study revealed that the strength of bone–titanium integration was considerably greater for implants with nanoparticles [26]. Therefore, avoiding the agglomeration of nanoparticles during the manufacturing process is important. Till date, several studies have reported that the addition of TiO_2_ nanoparticles to PEEK/n-TiO_2_ composites significantly enhanced the bioactivity of PEEK [27,28]. However, because of the high pressure and high temperature conditions required for molding in the traditional manufacturing procedures, the TiO_2_ nanoparticles do not remain well distributed and agglomerate into microparticles [27,28]. However, our PEEK/n-TiO_2_ composite was manufactured using the AM technology, which does not require high pressure; hence, most of the TiO_2_ particles distributed on the composite surface were still nanoparticles.

First, we tested the pore size and roughness of the composite using an atomic force microscope; however, the surface was too rough to test these two parameters. Therefore, a 3D profiler was used to detect the roughness, but still the pore size was too large for testing. Many gaps were clearly observed on the surface. Although the Ra values of the two samples were similar, the Rq and Rt values of the composite were higher than those of the PEEK group. This also indicates a higher roughness of the composite surface, which is consistent with the SEM results. We also observed that the Rt value of the composite was significantly higher than that of PEEK. Notably, the gaps parallel to the printing path in the PEEK sample were wider than those in the composite; thus, the melted composite filament showed better fluidity. The valleys perpendicular to the printing path result in a higher Rt value for the composite. This might be due to the higher fluidity of the melted composite filaments. Better fluidity would fill the gap generated in the previously printed layer.

SEM imaging also showed that the surface of the PEEK/n-TiO_2_ composite was rougher than that of the control PEEK. Generally, a rougher surface indicates better cell attachment [27], which might be another reason for the enhanced proliferation of MC3T3 cells, in addition to the TiO_2_ nanoparticles themselves. The SEM data also indicated that there was a significant difference in smoothness between the bottom (smoother) and surface (rougher) of the PEEK/n-TiO_2_ composite. We inferred that this might be due to the different temperatures of the print bed and print chamber or the different interfaces (solid and gas, respectively).

OCN is the most abundant non-collagenous protein in the bone matrix and plays a crucial role in the biomineralization process during osteogenic maturation [29]. COLI is involved in the formation of bone matrix and is synthesized and secreted by osteoblasts [30]. In this study, the incorporation of n-TiO_2_ significantly enhanced the expression of these two osteogenic markers and the other three markers (OPN, RUNX2, and ALP), as well as increased mineralization in MC3T3-E1 cells. This indicated that the 3D-printed PEEK/n-TiO_2_ composite reversed the positive osteogenesis of the TiO_2_ nanoparticles [16,31,32]. The stained mineralized nodules distributed on the PEEK or PEEK/n-TiO_2_ composite, where the cells were not well distributed, and a similar size of the well (culture plate) might be helpful to obtain a better cell distribution. Nevertheless, there was a significant difference in mineralization levels between the two groups. The osteointegration effect of the PEEK/n-TiO_2_ composite awaits further *in vivo* evaluation, which also requires the design of different pore sizes of the PEEK/n-TiO_2_ scaffold.

To determine the potential miRNA responsible for the improved properties of the composite, several related candidates were considered. miR-17 and miR-21 are both upregulated in MC3T3 cells cultured on glass coated with a TiO_2_ layer compared to those cultured on the uncoated substrate glass [20]. The expression changes of miR-154-3p, miR-154-5p, and miR-770-5p in hASCs cultured on TiO_2_-nanotube are consistent in both the proliferation and induction processes (vs cells cultured on pure Ti) [21]. Till date, the miRNA expression alterations induced by PEEK doped with n-TiO_2_, compared to pure PEEK, have been less investigated, and directly related miRNAs are still unknown. Although the control material was glass or Ti in the two studies [20,21], the miRNA expression change induced by the TiO_2_ layer or TiO_2_-nanotube still indicates the possible involvement of TiO_2_. Therefore, the aforementioned five miRNAs were selected for further investigation. miR-154-5p was downregulated in both proliferation and osteogenic differentiation processes in the composite group. Several studies have indicated that miR-154-5p plays a negative role in the proliferation of tumor cells [33,34]. Furthermore, miR-154-5p expression levels are increased in patients with type 2 diabetes mellitus and are negatively correlated with OCN [35]. Mechanical tension treatment causes the downregulation of miR-154-5p in adipose-derived mesenchymal stem cells and the forced expression of miR-154-5p inhibits osteogenic differentiation [36]. These studies suggest a negative role of miR-154-5p on cell proliferation and osteogenic differentiation. Consistently, our study also uncovered its negative regulatory role in the proliferation and osteogenic differentiation of MC3T3-E1 cells, which is an important mechanism for the improved properties of PEEK/n-TiO_2_.

One limitation of this study is that only a single PEEK/n-TiO_2_ composite with 30% n-TiO_2_ and no other composite with a different composition was investigated. Another limitation was that the downstream target gene of miR-154-5p was unknown, which will be investigated in future studies.

In summary, we successfully printed a PEEK/n-TiO_2_ composite with higher bioactivity than pure PEEK. Moreover, the composite retained the nanoscale size of TiO_2_ as well as the positive osteogenesis of the TiO_2_ nanoparticles, which provides a promising new solution to improve the osteointegration of PEEK. We also proved that the downregulation of miR-154-5p was an important mechanism for the improved properties of the PEEK/n-TiO_2_ composite.
